# Diversity characteristics of arbuscular mycorrhizal fungi communities in the soil along successional altitudes of Helan Mountain, arid, and semi-arid regions of China

**DOI:** 10.3389/fmicb.2023.1099131

**Published:** 2023-03-02

**Authors:** Peixuan Yan, Hui Hou, Yingze Lv, Haiying Zhang, Jia Li, Leilei Shao, Qinmi Xie, Yongliang Liang, Jingyao Li, Xilu Ni

**Affiliations:** ^1^College of Agriculture, Ningxia University, Yinchuan, China; ^2^Breeding Base for State Key Laboratory of Land Degradation and Ecological Restoration in Northwest China, School of Ecological and Environment, Ningxia University, Yinchuan, China; ^3^Key Laboratory of Restoration and Reconstruction of Degraded Ecosystems in Northwestern China of Ministry of Education, Ningxia University, Yinchuan, China; ^4^Ningxia Helan Mountain Forest Ecosystem Orientation Observation Research Station, Yinchuan, China

**Keywords:** arbuscular mycorrhizal fungi, community structure, altitude, soil factor, Helan Mountain

## Abstract

**Introduction:**

Arbuscular mycorrhizal fungi (AMF) perform a vital role in terrestrial ecosystems.

**Methods:**

To investigate the diversity of AMF communities on the western slope of Helan Mountain at different altitudes and their influence factors, high-throughput sequencing was used to study the structure and diversity of soil AMF communities under different environments and their interrelationships between AMF and environmental factors.

**Results:**

The results revealed that there were significant differences (*p* < 0.05) in the physical and chemical properties of the soil along the different altitudes. A total of 1,145 OTUs were obtained by high-throughput sequencing, belonging to 1 phylum, 4 class, 6 orders, 13 families, 18 genera and 135 species, with the dominant genus being *Glomus*, which accounted for 75.27% of the relative abundance of the community. Soil AMF community structure was shown to be variable at the generic level according to NMDS analysis. Correlation analysis showed that soil pH, water content (WC), organic matter (OM), available K, available P and N were significantly correlated with AMF community diversity and species abundance (*p* < 0.05, *p* < 0.01). Based on redundancy analysis (RDA) and Monte Carlo test results, soil pH, WC and OM had highly significant effects (*p* < 0.01) on AMF community diversity and species abundance.

**Discussion:**

This study investigates the relationship between AMF community structure and diversity and soil physicochemical properties at different elevations on the western slope of Helan Mountain, which is of great significance to the study of the Helan Mountain ecosystem.

## 1. Introduction

Arbuscular mycorrhizal fungi (AMF) are mycorrhizal fungi that exist in the inter-rooted soil and can form symbiotic relationships with 80% of terrestrial vascular plants (Davison et al., 2015; Shi et al., 2020) and are widely found in various terrestrial ecosystems, playing an irreplaceable role in maintaining and improving vegetation communities, soil conditions and stabilizing ecosystem functions, and are an important component of terrestrial ecosystems (Elhindi et al., 2018; Qiang et al., 2019). It has been found that AMF can regulate the composition of soil inter-root microbial communities, soil structure and nutrient cycling (Cheng et al., 2012; Veresoglou et al., 2012); and after symbiosis with plants, it can not only improve the uptake of N, P and other nutrients by plants, but also enhance the resistance of plants to drought, salinity and pests and diseases, and promote plant growth (Auge, 2001; Garg and Chandel, 2011; Hodge and Storer, 2015). AMF communities are also influenced by a variety of environmental factors, such as altitude, plant communities, soil physicochemical properties and climatic factors. Especially, soil C, N, and P content and host plant species have a significant impact on the diversity and abundance of AMF communities (Kruger et al., 2017; Ezeokoli et al., 2019). Therefore, research on the relationship between AMF communities and environmental factors contributes to a better understanding of terrestrial ecosystems.

In mountain ecosystems, environmental factors can change considerably at smaller scales with altitude, causing differences in plant and animal communities between different altitudes (Deng et al., 2020; Zanzottera et al., 2020). Both changes in plant communities and soil characteristics affect the community structure of microorganisms in the soil (Li et al., 2018). Vieira et al. (2019) showed that AMF community diversity decreased with elevation increased in the zone of temperate climates. However, some studies have shown that AMF community diversity is independent or negatively correlated with elevations (Coutinho et al., 2015; Shen et al., 2015). In addition, slope orientation, an important topographic factor, and soil physicochemical properties and vegetation type differences between shady and sunny slopes (Sun et al., 2019; Guo et al., 2022) may affect AMF community structure. In conclusion, the different vegetation types, temperature, light and soil characteristics that different altitudes have can affect the AMF community. In recent years, there have been many achievements in the study of AMF. Among them, Dong et al. (2023) showed that drought conditions can have an impact on AMF growth and symbiosis with plants. Zhang et al. (2021) showed the effect of altitude on AMF community in Qinling Mountain. Zhao et al. (2019) showed that plant community succession has an important influence on AMF community. Liu et al. (2020) showed the influence of season and soil on AMF community in mountainous areas. The results of these studies show that soil and vegetation are the main environmental factors affecting AMF community, and that climate, seasonality and precipitation all indirectly affect AMF community through soil, but the types, magnitude and trends of the effects of soil factors on AMF community vary from different studies and need to be explored in depth.

Helan Mountain, located in the northwest of Ningxia, is an important natural geographical boundary and the dividing line between grassland and desert in northwest China, and plays the role of an important ecological barrier with a unique ecosystem. The vegetation types vary significantly along the successional altitudes, and the vegetation types are in order of desertification grassland, mountain scrub, sparse forest grassland, mountain coniferous forest and subalpine scrub meadow, etc., along the altitude rises (Wu et al., 2021). Vegetation diversity is often closely related to AMF community diversity and there is a positive interaction between them. The rich AMF resources contained in different elevations of Helan Mountain may play an important role in maintaining and protecting the stability of different vegetation ecosystems in Helan Mountain, but there is still a gap in research on the AMF communities in Helan Mountain.

Therefore, this study uses high-throughput sequencing technology to study the diversity of AMF communities in rhizosphere soil at different altitudes on the western slope of Helan Mountain, and to analyze the correlation between the physical and chemical properties of rhizosphere soil and the structural characteristics of AMF communities on the western slope of Helan Mountain, to provide a theoretical basis for maintaining the stability and development of Helan Mountain ecosystems and exploring the role of AMF communities in terrestrial ecosystems.

## 2. Materials and methods

### 2.1. Study area

Surveyed sites are located on the western slope of Helan Mountain in Alxa Left Banner, Inner Mongolia Autonomous Region. It is located in the eastern edge of the Alxa Plateau, the west side of the Yinchuan Plain (between 38°27′-39°30′N and 105°41′-106°41′E), with a typical continental climate and mountainous climatic characteristics. The average annual temperature is 8.6°C, the annual precipitation is 200–400 mm, mainly concentrated in May–September, and the annual evaporation is high, reaching over 2,000 mm. The main plant species in the study area are *Agropyron mongolicum* (Keng), *Stipa breviflora* (Griseb), *Prunus mongolica* (Maxim), *Caragana stenophylla* (Pojark), *Ulmus glaucescens* (Franch), *Juniperus rigida* (Siebold & Zucc), *Pinus tabuliformis* (Carriere), and *Picea crassifolia* (Kom), etc.

### 2.2. Experimental design

Based on the field survey, eight altitudes (ALT) were selected along the altitude gradient according to different community types on the western slope of Helan Mountain from 1800 to 2,750 m above sea level in the summer of 2021, and three sample plots were set up within each vegetation zone with a sample area of 20 m × 20 m. The soil samples were collected by digging up 5 plant rhizosphere soil samples using a 4-cm diameter soil sampler according to the five-point sampling method, mixing them well, placing them in sterilized bags and storing them in a refrigerated incubator. The soil was taken back to the laboratory and stored in a refrigerator at −80°C for the determination of soil AMF community diversity and naturally dried and sieved for the determination of soil physical and chemical properties (Table 1).

**Table 1:** Basic information about the sample site.

Plot	Type	Altitude	Major plant species
PI	*Picea crassifolia* Forests	2,643	*Picea crassifolia* (Kom)
		2,638	
		2,638	
SU	Subalpine Meadow	2,635	*Kobresia pygmaea* (C. B. Clarke), etc
		2,635	
		2,635	
CO	Coniferous Mixed Forest	2,360	*Picea crassifolia* (Kom) *Pinus tabuliformis* (Carriere), etc
		2,360	
		2,360	
CB	Coniferous and Broad-Leaved Mixed Forest	2,190	*Populus davidiana* (Dode) *Pinus tabuliformis* (Carriere) *Picea crassifolia* (Kom), etc
		2,183	
		2,160	
PF	Pinus Forest	2,173	*Pinus tabuliformis* (Carriere)
		2,129	
		2,123	
SH	Shrub	2,110	*Prunus mongolica* (Maxim) *Caragana stenophylla* (Pojark), etc
		2,110	
		2,110	
UL	*Ulmus glaucescens* Forests	1,910	*Ulmus glaucescens* (Franch)
		1,910	
		1,905	
GR	Grass	1,856	*Agropyron mongolicum* (Keng) *Stipa breviflora* (Griseb), etc
		1,856	
		1,856	

### 2.3. Measurement items and methods

Soil pH was determined using pH meter with a 5:1 ratio of water to soil; water content (WC) was determined using the drying method; organic matter (OM) was determined using the potassium dichromate dilution calorimetric method; total nitrogen (TN) was determined using the semi-micro Kjeldahl method; alkali-hydrolyzable nitrogen (AN) was determined by alkaline diffusion; total phosphorus (TP) was determined by HClO_4_-H_2_SO_4_ digestion with molybdenum-antimony colorimetry; available phosphorus (AP) was determined by NaHCO_3_ leaching with molybdenum-antimony colorimetry; available potassium (AK) was determined by NH_4_OAc extraction and flame photometric method.

Soil AMF community diversity was sent to Majorbio Bio-Pharm Technology Co. Ltd (Shanghai, China) for determination, and soil AMF community DNA was extracted using the MP kit, using AMV4-5NF/AMDGR primers (Sato et al., 2005) with primer sequences of 5′- AAGCTCGTAGTTGAATTTCG-3′, 5’-CCCAACTATCCCTATTAATCAT-3′, and PCR amplification of the 18S rRNA gene was performed. PCR amplification cycling conditions were as follows: initial denaturation at 95°C for 3 min, followed by 27 cycles of denaturing at 95°C for 30 s, annealing at 55°C for 30 s and extension at 72°C for 45 s, and single extension at 72°C for 10 min, and end at 4°C.

Library construction of PCR products using TruSeq^TM^ DNA Sample Prep Kit: (1) Addition of official Illumina splice sequences to the outer end of the target region by PCR; (2) Recovery of PCR products by gel cutting using a gel recovery kit; (3) Elution in Tris–HCl buffer and detection by 2% agarose electrophoresis; (4) Denaturation by sodium hydroxide to produce single-stranded DNA fragments. Paired-end sequencing was performed on an Illumina MiSeq PE300 platform (Illumina, San Diego, United States). The raw sequencing reads were deposited into the NCBI Sequence Read Archive (SRA) database (Accession Number: PRJMNA932928).

Using UPARSE (Stackebrandt and Goebel, 1994; Edgar, 2013) software^1^ (version 7.1), OTUs were classified at 97% sequence similarity and the RDP classifier Bayesian algorithm was used to taxonomically analyze representative sequences of OTUs at 97% similarity level by maarjam20220506/AM species database for comparison and to obtain taxonomic information.

### 2.4. Data analysis

Data were collated using EXCEL 2019 and one-way ANOVA (One-way ANOVA) and Duncan’s test (Duncan’s test) were used to analyze the differences in soil physicochemical properties between vegetation types using SPSS22 (*p* < 0.05), and one-tailed significance test was used to test Person between different variables. Correlation analysis was performed, and differences between AM fungal communities were analyzed using non-metric multidimensional scaling analysis (NMDS) and analysis of similarity test (ANOSIM), with *r* and *p* values associated with NMDS calculated based on 999 permutations of ANOSIM. Origin 2019 was used for mapping. Soil AMF community diversity, β-diversity and structure were analyzed and mapped using the computing platform of Shanghai Meiji Biomedical Technology Company Limited and the R language “Vegan” package.

The AMF community diversity was analyzed using the Shannon index, the Simpson index, the Chao1 index and the ACE index.

#### 2.4.1. Shannon-Wiener index

The formula is calculated as:


$$H_{Shannon}=-\overset{S_{obs}}{\underset{i=1}{\sum }}\frac{n_{i}}{N}\ln\frac{n_{i}}{N}$$

#### 2.4.2. Simpson index

The formula is calculated as:


$$D_{Simpson}=\frac{\sum_{i=1}^{S_{obs}}n_{i}\left(n_{i}-1\right)}{N\left(N-1\right)}$$

Where, Sobs is the number of OTUs observed in practice; n_i_ is the number of sequences contained in the i-th OTU; and N is the number of all sequences.

#### 2.4.3. Chao1 index

The formula is calculated as:


$$S_{Chao1}=S_{obs}+\frac{n_{1}\left(n_{1}-1\right)}{2\left(n_{2}-1\right)}$$

Where, S_Chao1_ is the estimated number of OTUs; n_1_ is the number of OTUs containing only one sequence; n_2_ is the number of OTUs containing only two sequences.

#### 2.4.4. ACE index

The formula is calculated as:


$$f\left(x\right)=\left\{\begin{array}{c} S_{abund}+\frac{S_{rare}}{C_{ACE}}+\frac{n_{1}}{C_{ACE}}{\hat{\gamma }}_{ACE}{}^{2},for\;\gamma_{ACE}<0.80\\ S_{abund}+\frac{S_{rare}}{C_{ACE}}+\frac{n_{1}}{C_{ACE}}{\tilde{\gamma }}_{ACE}{}^{2},for\;{\hat{\gamma }}_{ACE}\geq 0.80 \end{array}\right.$$

Among these:


$$N_{rare}=\overset{abund}{\underset{i=1}{\sum }}in_{i},C_{ACE}=1-\frac{n_{1}}{N_{rare}}$$

$${\hat{\gamma }}^{2}{}_{ACE}=\max\left[\frac{S_{rare}}{C_{ACE}}\frac{\sum_{i=1}^{abund}i\left(i-1\right)n_{i}}{N_{rare}\left(N_{rare}-1\right)}-1,0\right]$$

$${\tilde{\gamma }}^{2}{}_{ACE}=\max\left[{\tilde{\gamma }}^{2}{}_{ACE}\left\{1+\frac{N_{rare}\left(1-C_{ACE}\right)\sum_{i=1}^{abund}i\left(i-1\right)n_{i}}{N_{rare}\left(N_{rare}-C_{ACE}\right)}\right\},0\right]$$

Where, S_rare_ is the number of OTUs containing “abund” sequences or less than “abund”; S_abund_ is the number of OTUs with more than “abund” sequences; abund is the threshold of “dominant” OTUs, default is 10.

## 3. Results

### 3.1. Physico-chemical properties of soil at different altitudes

As can be seen from Table 2, there were significant differences in soil physicochemical properties between different altitudes (*p* < 0.05). WC, OM, AK and AN increased with the increasing altitude, and the maximum values occurring in the high-altitude region (they are 14.95, 11.70%, 54.27 mg/kg, 96.73 mg/kg respectively). Both pH and TP increased and then decreased with increasing altitude, with the maximum values occurring in the mid-altitude region (they are 8.20, 213.81 mg/kg respectively), where the pH varied from 7.94 to 8.20, indicating that the soil on the western slope of Helan Mountain is alkaline. TN and AP decreased and then increased with increasing altitude, with the maximum TN value occurring at high-altitude areas (202.61 mg/kg) and the maximum AP value at low-altitude areas (55.37 mg/kg). Overall, the nutrient content of the soil increases with altitude.

**Table 2:** Physico-chemical properties of soil at different altitudes.

PLOT	WC/%	pH	TN/(mg/kg)	AN/(mg/kg)	TP/(mg/kg)	AP/(mg/kg)	OM/%	AK/(mg/kg)
SU	14.95 ± 1.77a	7.95 ± 0.03d	113.25 ± 1.89bc	60.77 ± 16.79bc	202.20 ± 6.42b	26.17 ± 2.41c	11.70 ± 0.55a	54.27 ± 3.23a
PI	7.64 ± 2.12b	7.94 ± 0.04d	202.61 ± 2.47a	96.73 ± 16.44a	127.14 ± 5.13d	49.14 ± 2.64ab	7.04 ± 0.46b	31.13 ± 5.40b
CO	7.06 ± 2.17b	8.05 ± 0.06c	104.58 ± 8.15c	55.13 ± 2.14bc	130.33 ± 3.71d	50.43 ± 8.3ab	4.24 ± 0.61d	27.80 ± 6.91b
CB	3.80 ± 1.14 cd	8.20 ± 0.04a	103.51 ± 7.55c	65.91 ± 1.33b	213.81 ± 6.06a	44.80 ± 2.98b	5.83 ± 0.70c	45.67 ± 4.54a
PF	7.65 ± 2.53b	8.06 ± 0.03c	54.35 ± 12.72e	23.60 ± 9.78d	144.57 ± 3.18c	44.01 ± 4.73b	4.98 ± 0.69 cd	32.17 ± 1.94b
SH	5.41 ± 1.68bc	8.09 ± 0.02bc	78.20 ± 9.46d	45.28 ± 3.79c	132.33 ± 1.90d	43.95 ± 1.27b	5.68 ± 0.11c	28.33 ± 3.16b
UL	2.01 ± 0.32d	8.09 ± 0.04bc	121.21 ± 10.98b	48.83 ± 14.00dbc	111.94 ± 7.93e	45.28 ± 4.95b	2.67 ± 0.19e	31.87 ± 3.46b
GR	1.49 ± 0.36d	8.14 ± 0.02ab	88.10 ± 0.54d	10.25 ± 5.44d	106.16 ± 9.47e	55.37 ± 6.88a	0.99 ± 0.23f	28.73 ± 8.81b

Different lowercase letters indicate significant differences between treatments (*P* < 0.05). Values are means ± standard errors (SE; *n* = 3).

### 3.2. Soil arbuscular mycorrhizal fungi community diversity at different altitudes

**Figure 1:**
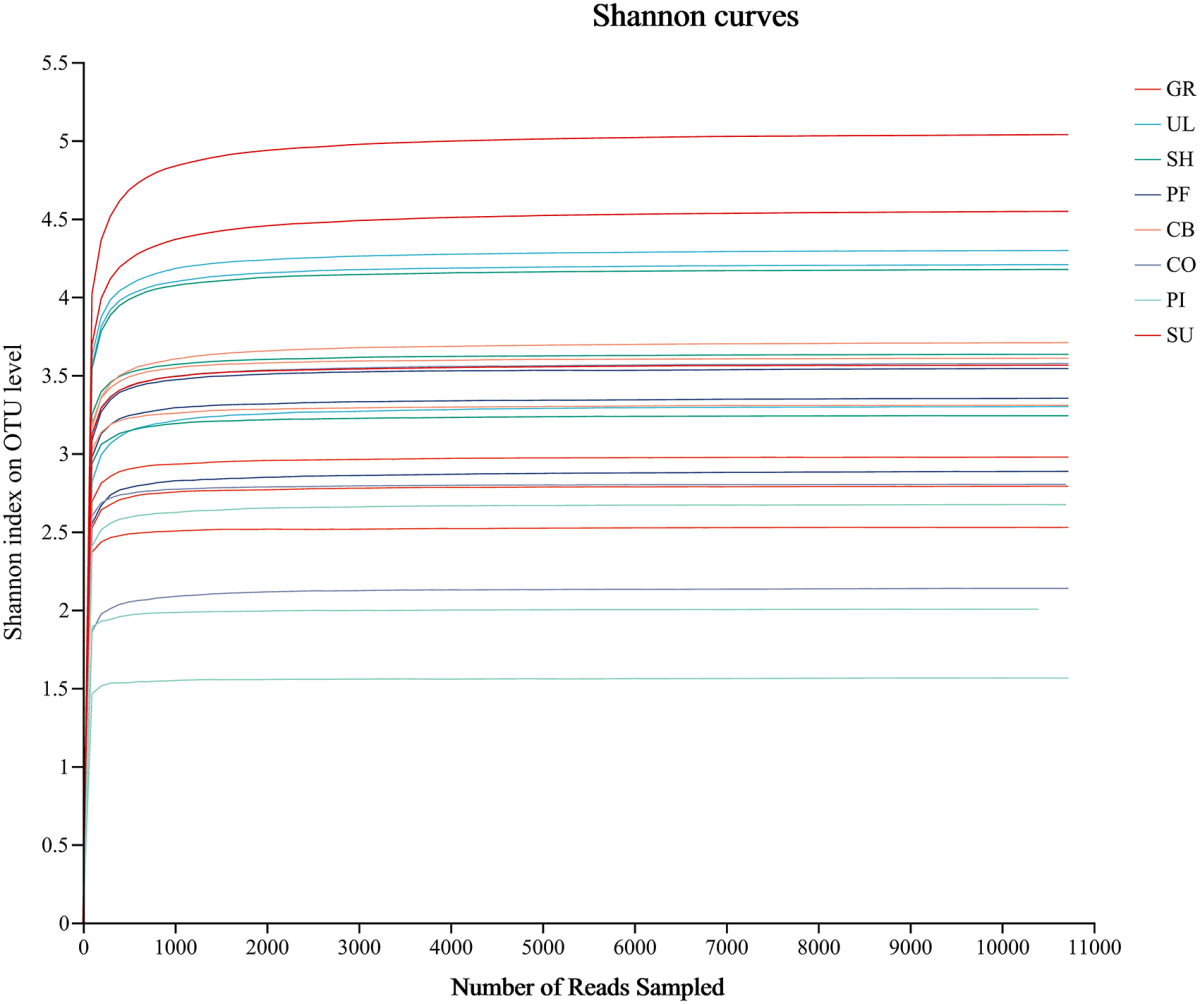
AMF community dilution curves for soil at different elevations.

As can be seen from Figure 1, the dilution curve flattens out as the number of sequenced bars increases, indicating that the amount of sample sequencing data is large enough to reflect the true situation of the AMF community in the soil and that subsequent data analysis can be carried out.

A total of 2,158,539 valid sequences with a total base number of 466,973,432 bp and an average sequence length of 216 bp were obtained from the AMF assay for each sample. The coverage of each sample was above 99%, indicating that the sequencing results are representative of the AMF diversity in the samples. As can be seen from Table 3, there were significant differences in soil AMF community diversity between elevations (*p* < 0.05). The soil AMF community of SU had the largest Shannon index and the smallest Simpson index, while the soil AMF community of PI had the smallest Shannon index and the largest Simpson index. The maximum values of the ACE and Chao1 indices are at SU and the minimum values are at GR and PI, respectively. Overall, the diversity of the soil AMF community increases with altitude.

**Table 3:** AMF diversity indices for soils at different altitudes.

PLOT	Shannon	Simpson	ACE	Chao1	Coverage
SU	4.38 ± 0.75a	0.03 ± 0.02b	395.02 ± 144.70a	395 ± 145a	0.9954 ± 0.0009d
PI	2.08 ± 0.56d	0.21 ± 0.10a	140.13 ± 90.58 cd	96 ± 35c	0.9981 ± 0.0008ab
CO	2.84 ± 0.72 cd	0.16 ± 0.15ab	146.48 ± 66.25 cd	144 ± 61c	0.9979 ± 0.0010ab
CB	3.54 ± 0.21abc	0.06 ± 0.003b	240.62 ± 21.54bc	221 ± 49bc	0.9961 ± 0.0004 cd
PF	3.26 ± 0.34bc	0.10 ± 0.04ab	192.178 ± 26.89bcd	187 ± 24bc	0.9969 ± 0.0006bc
SH	3.68 ± 0.47abc	0.05 ± 0.02b	216.06 ± 72.20bcd	208 ± 69bc	0.9971 ± 0.0008bc
UL	3.93 ± 0.55ab	0.07 ± 0.06b	293.93 ± 13.80ab	296 ± 27ab	0.9956 ± 0.0009 cd
GR	2.76 ± 0.23 cd	0.11 ± 0.02ab	99.31 ± 26.86d	102 ± 26c	0.9986 ± 0.0007a

Different lowercase letters indicate significant differences between treatments (*P* < 0.05). Values are means ± standard errors (SE; *n* = 3).

### 3.3. Taxonomic composition and distribution of arbuscular mycorrhizal fungi in soil at different altitudes

The results of the high-throughput sequencing data analysis showed that the comparison with the maarjam20220506/AM species database revealed a total of one phylum, four class, six orders, 13 families, 18 genera and 135 species at different altitudes.

**Figure 2:**
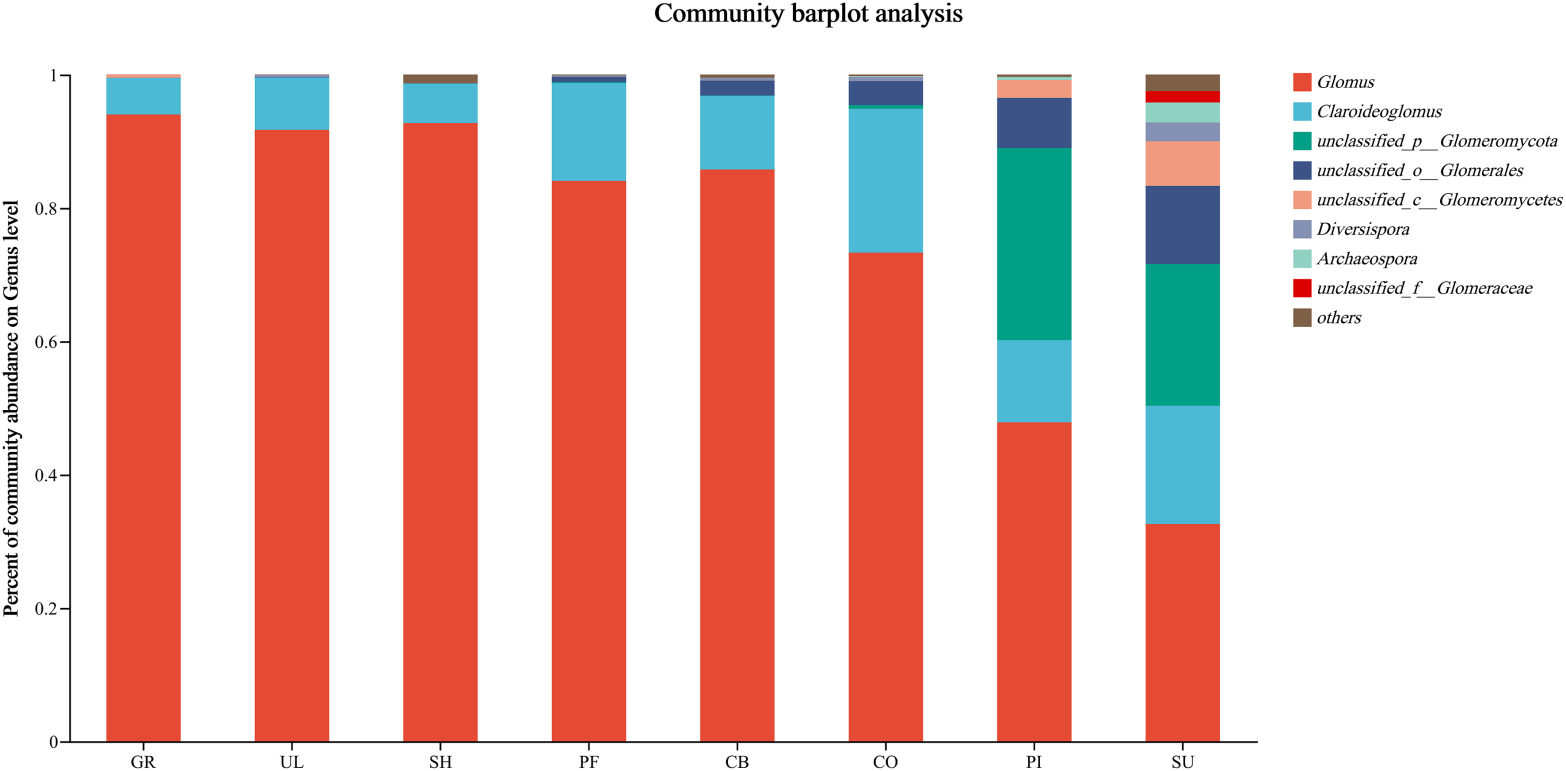
Genus level composition of AMF communities in soil at different altitudes.

**Figure 3:**
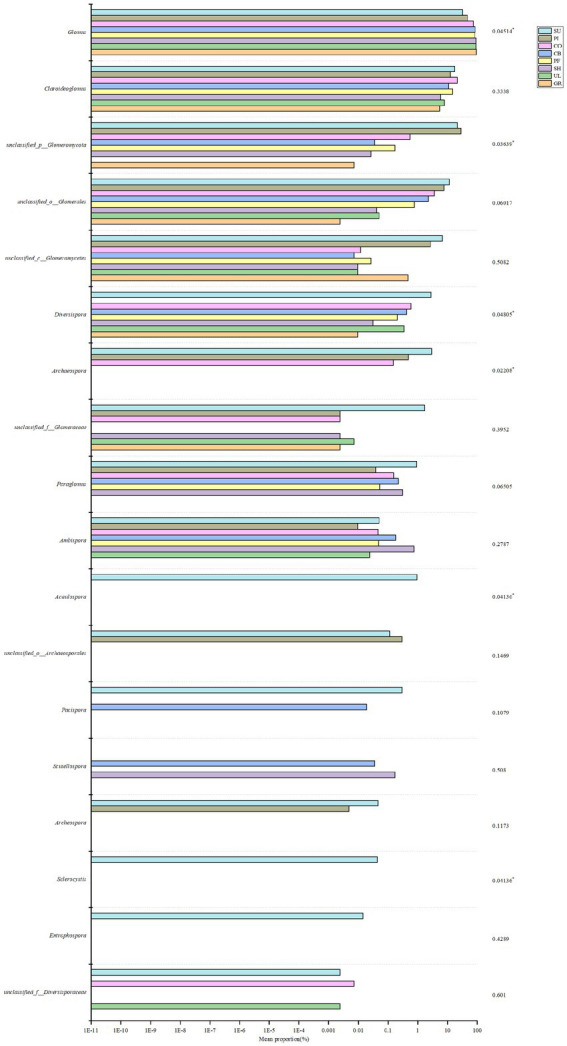
Significance test for differences in AMF species at different altitudes. *represent significant correlations at the 0.05, **represent significant correlations at the 0.01, and ***represent significant correlations at the 0.001.

According to Figure 2, the relative abundance of soil AMF at the genus level varied between samples. The relative abundance of *Glomus* was the highest in all samples, averaging 75.27%, reaching a maximum relative abundance in GR (94.04%), and exceeding 90% in GR, UL and SH, indicating that *Glomus* is the dominant species in the soil AMF community on the western slope of Helan Mountain. Analysis of species differences at different sample subordinate levels showed (Figure 3) that *Acaulospora*, *Sclerocystis*, and *Entrophospora* were only found in the SU. *Glomus*, *unclassified_p__Glomeromycota*, *Diversispora*, *Archaeospora*, *Acaulospora*, and *Sclerocystis* were significantly different in different samples (*p* < 0.05).

**Figure 4:**
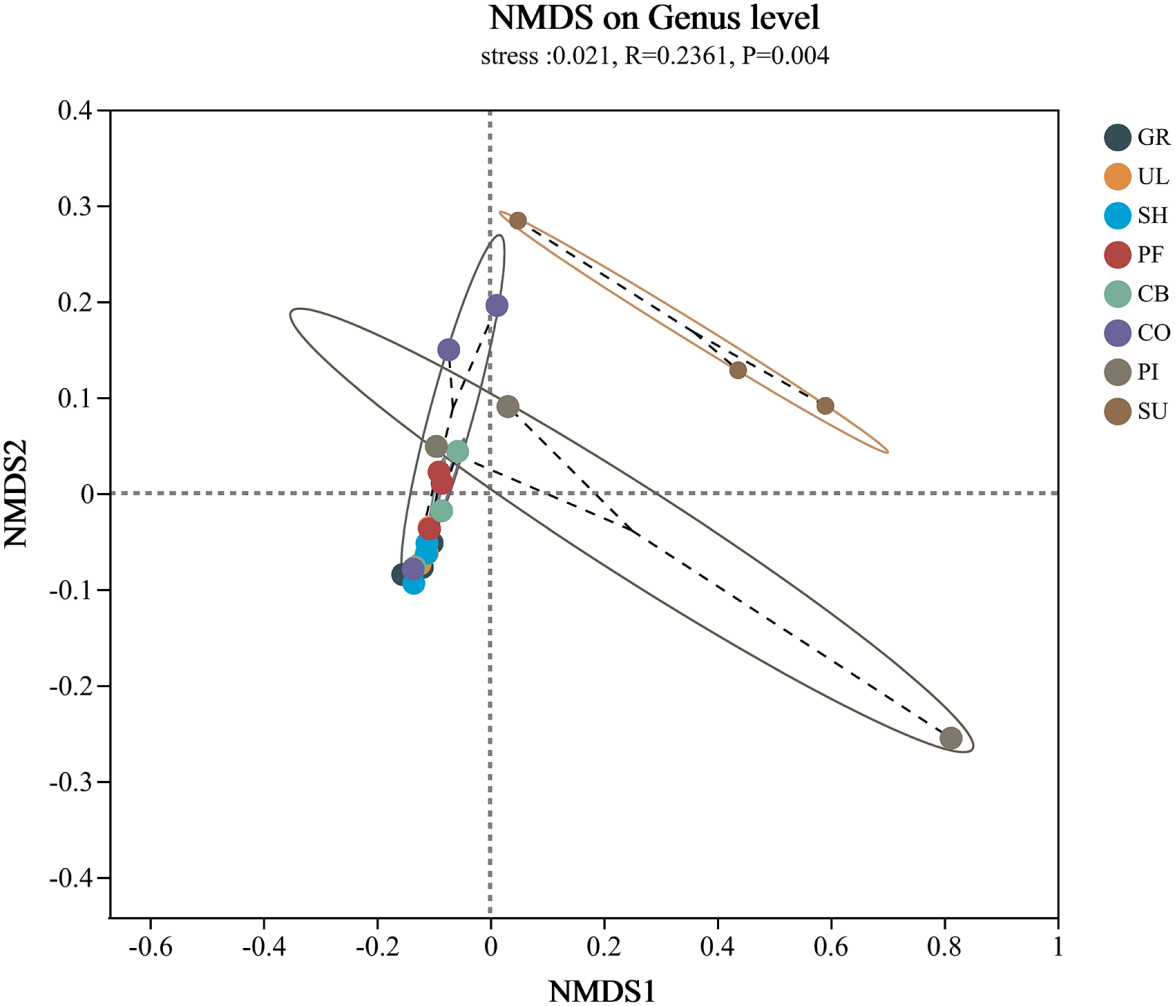
NMDS analysis of the β-diversity of soil AMF communities at different altitudes.

**Figure 5:**
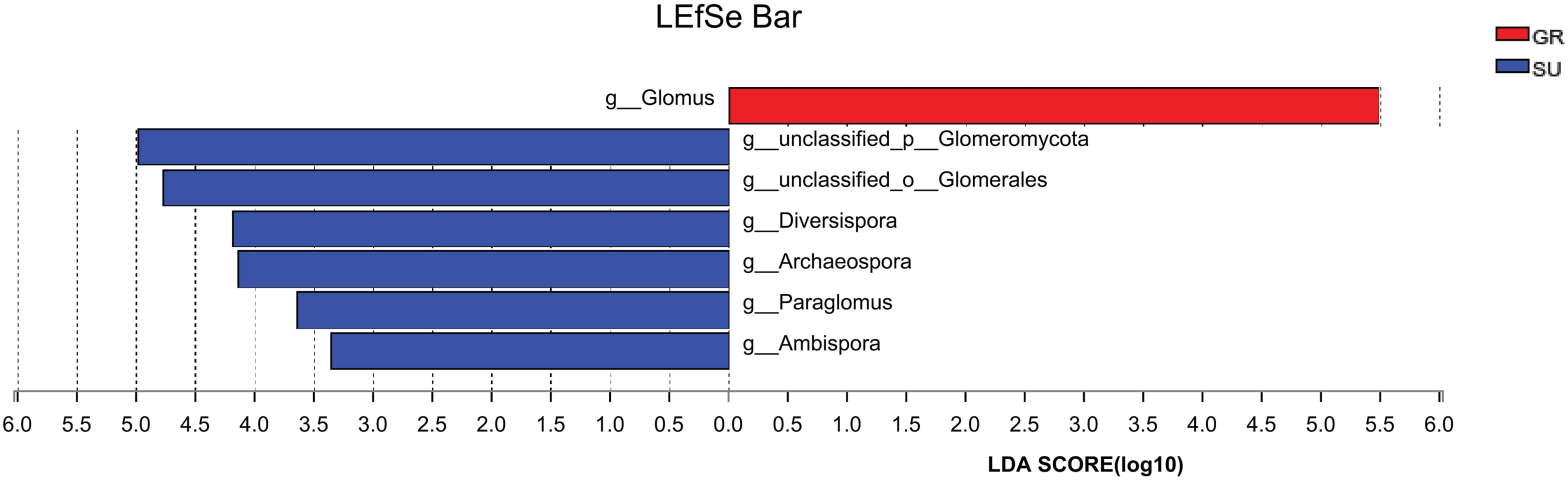
LEfSe analysis of GR and SU.

NMDS of soil AMF community structure based on genus level and bray_curtis distance calculations showed (Figure 3) that the PI group was relatively more dispersed within its group, indicating poor intra-group repeatability; the remaining samples were relatively more concentrated within their group, indicating good inter-replicate similarity within the remaining sample groups (Figure 4). The larger area of overlap between the GR, UL, and SF groups at low-altitude indicates that there is less difference in community structure between the three samples, whereas there is no area of overlap between PI and SC at high-altitude, indicating that there is a greater difference in community structure between the two samples and a greater difference in community structure between the low and high altitudes. LEfse analysis was further carried out for the two groups of samples with the greatest differences (GR and SU; Figure 5), of which the abundance of *Glomus* was significantly higher in GR than in SU, and the abundance of *unclassified_p__Glomeromycota*, *unclassified_o__Glomerales*, *Diversispora*, *Archaeospora*, *Paraglomus,* and *Ambispora* was significantly higher in SU than in GR.

### 3.4. Correlation analysis between the physico-chemical properties of soil and the diversity of arbuscular mycorrhizal fungi communities at different altitudes

The correlation analysis showed (Table 4) that the ALT, WC, OM, and AK were highly significantly positively correlated with the Shannon Index, ACE Index and Chao1 Index, and highly significantly negatively correlated with the Simpson Index. The pH was highly significantly negatively correlated with the Shannon index, significantly positively correlated with the ACE and Chao1 indices and highly significantly positively correlated with the Simpson index. The AN was significantly negatively correlated with the Simpson Index and significantly positively correlated with the ACE Index. The TP was significantly and positively correlated with the Shannon index, the ACE index and the Chao1 index. The AP was highly significantly negatively correlated with the Shannon and Chao1 indices, significantly negatively correlated with the AVCE index and highly significantly positively correlated with the Simpson index.

**Table 4:** Person correlation analysis between soil AMF community diversity and soil physicochemical properties.

	ALT	Shannon	Simpson	ACE	Chao1
ALT		0.741**	−0.770**	0.525**	0.599**
WC	0.792**	0.739**	−0.713**	0.582**	0.716**
TN	0.566**	0.237	−0.267	0.283	0.184
pH	−0.738**	−0.542**	0.571**	−0.382*	−0.410*
AN	0.712**	0.329	−0.391*	0.357*	0.309
TP	0.441*	0.418*	−0.331	0.512*	0.483*
AP	−0.451*	−0.673**	0.628**	−0.362*	−0.633**
OM	0.834**	0.784**	−0.739**	0.648**	0.788**
AK	0.414*	0.671**	−0.647**	0.528**	0.655**

* and ** represent significant correlation at 0.05 and 0.01 levels, respectively.

**Figure 6:**
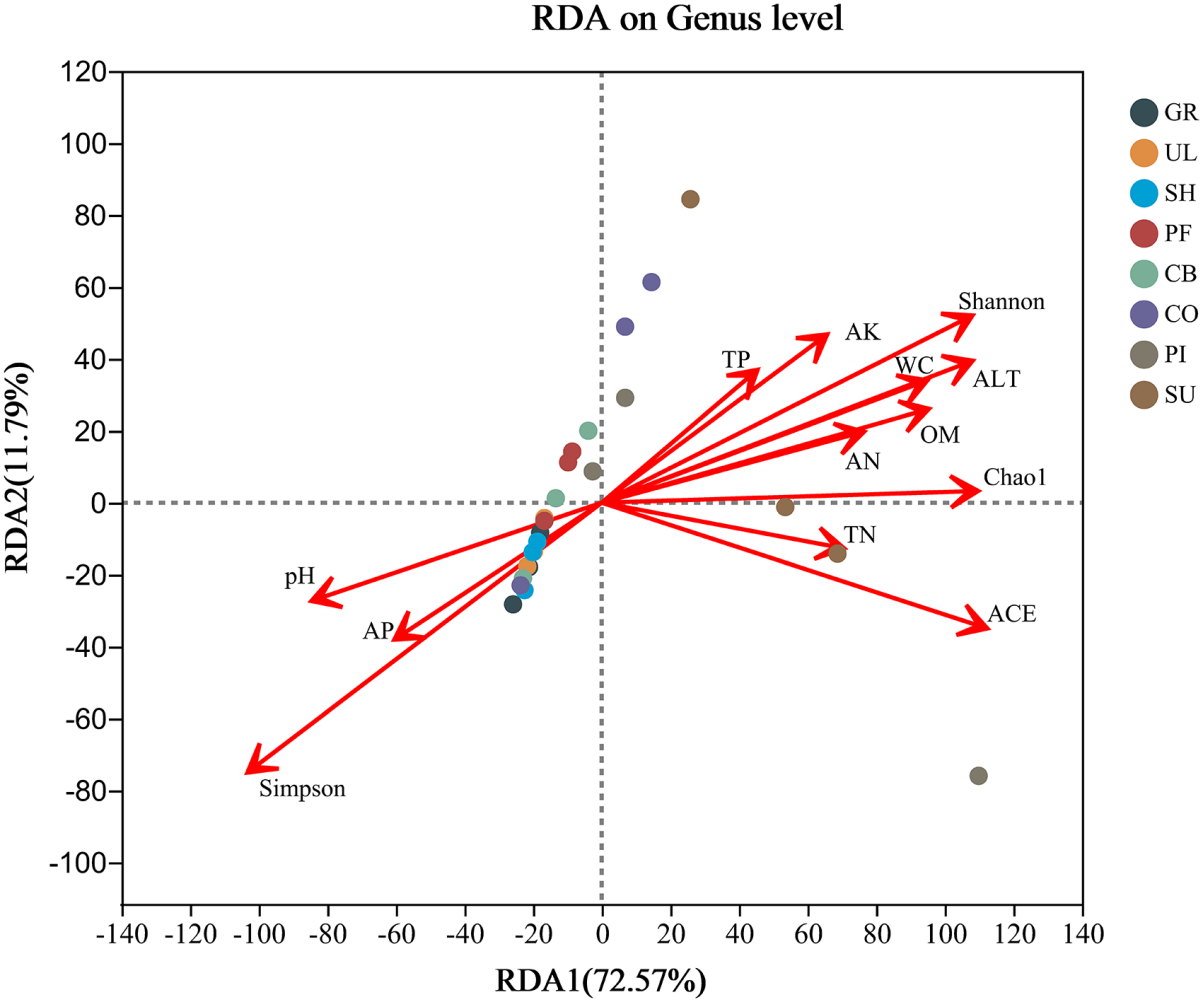
RDA between AMF diversity and physico-chemical properties of soil.

The RDA showed (Figure 6) that the Shannon index, ACE index and Chao1 index were positively correlated with ALT, AK, WC, OM, TP, AN and TN, and negatively correlated with pH and AP, with ALT having the greatest effect on the structure of the AMF community. According to Monte Carlo tests, ALT (*r*^2^ = 0.6623, *p* = 0.001), WC (*r*^2^ = 0.5036, *p* = 0.002), OM (*r*^2^ = 0.4820, *p* = 0.004) and pH (*r*^2^ = 0.3888, *p* = 0.006) had significant effects on AMF community diversity and species abundance. The first ordination axis explained 72.57% of the variation in AMF community composition and the second ordination axis explained 11.79% of the variation in AMF community composition. The first ordination axis explains most of the variation in soil AMF community diversity and richness at different elevations on the western slope of Helan Mountain.

**Figure 7:**
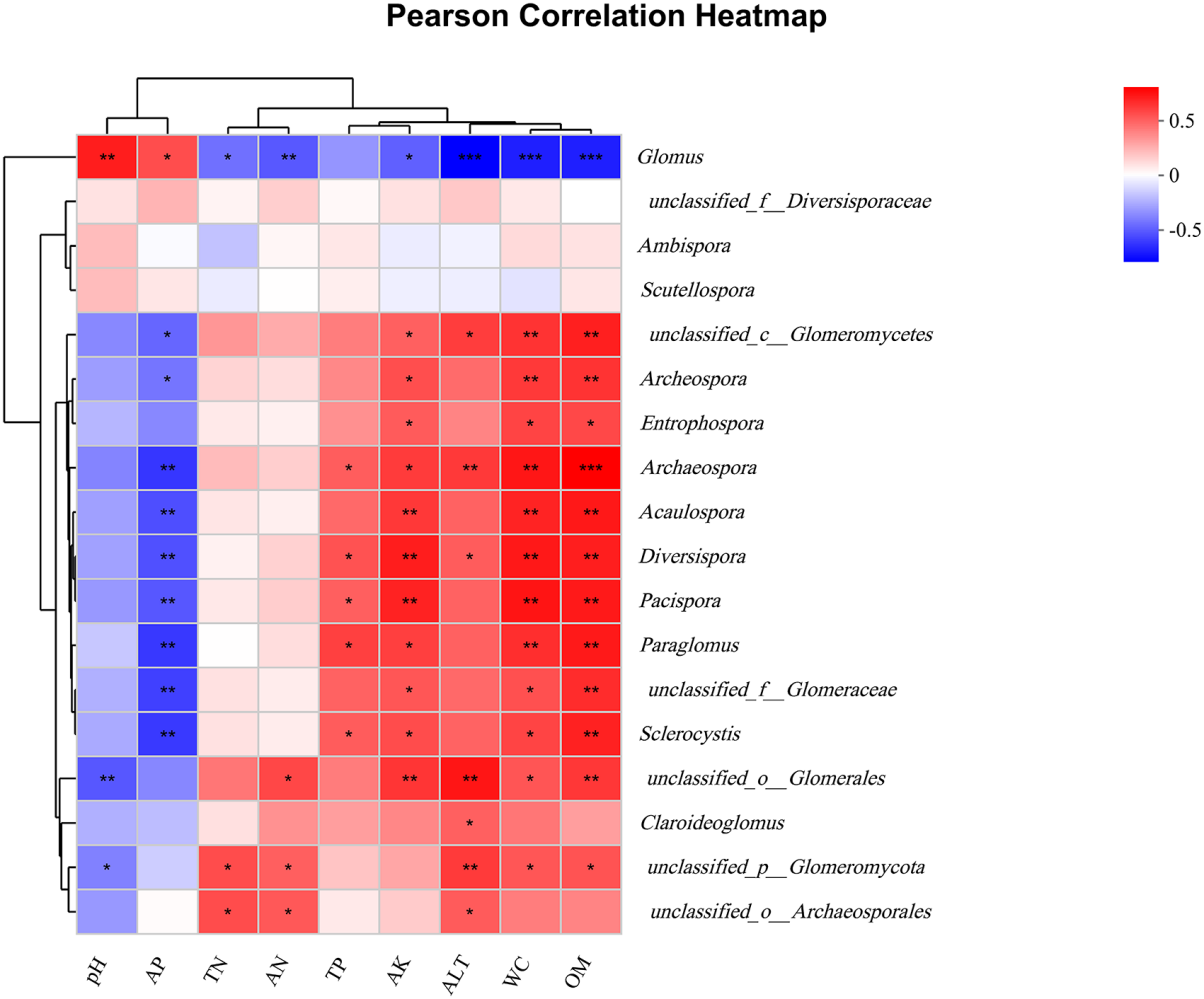
Heatmap of Person correlation between AMF community genus level and physico-chemical properties of soil. *represent significant correlations at the 0.05, **represent significant correlations at the 0.01, and ***represent significant correlations at the 0.001.

According to the results of Heatmap plots of environmental factors and soil AMF genus levels (Figure 7), soil physicochemical properties and altitude have different effects on each genus. *Glomus* was most affected by physico-chemical properties of soil and altitude, with *Glomus* being significantly negatively correlated with ALT, WC, OM, and AN, highly significantly positively correlated with pH, significantly negatively correlated with AK and TN, and significantly positively correlated with AP. The pH was highly significantly negatively correlated with *unclassified_o__Glomerales* and significantly positively correlated with u*nclassified_p__Glomeromycota*. *Acaulospora, Diversispora,* and *Pacispora*were highly significantly positively correlated with OM and AK, and highly significantly negatively correlated with AP. In addition, soil physicochemical properties and elevation had no significant effect on *Ambispora*, *unclassified_f__Diversisporaceae* and *Scutellospora*.

## 4. Discussion

### 4.1. Characteristics of changes in soil arbuscular mycorrhizal fungi community structure at different altitudes

A total of 1,418 AMF OTUs were obtained in this study, indicating that AMF species are abundant and widely distributed on the western slope of Helan Mountain, and are an important part of the ecosystem. The results of this study show that *Glomus* is the dominant genus in the soil AMF community on the western slope of Helan Mountain, which is consistent with the results of most studies on AMF communities in temperate mountainous areas (Zhang et al., 2022a,b), because *Glomus* contains a lots of species and can produce a large number of mycelial fragments and spores, allowing it to spread and colonize plant roots more widely and with greater environmental adaptability (Vandenkoornhuyse et al., 2002; Zhao et al., 2017). In the present study, the relative abundance of *Glomus* was highly significantly negatively correlated with altitude, which is consistent with the findings of Haug et al. (2019), while most other AMF genera were positively correlated with altitude. Several studies have shown that *Glomus* can enhance plant stress tolerance and biomass under drought conditions (Nacoon et al., 2021); appropriate increases in temperature and precipitation can enhance the photosynthetic efficiency of the host plant, allowing the plant to provide more photosynthetic products to AMF and promote mycelial and spore development (Zhao et al., 2022). As altitude increases, on the one hand, increased precipitation may make host plants less dependent on *Glomus*; on the other hand, lower temperatures limit the development of AMF mycelium and spores, which may have a greater impact on *Glomus* than other AMF genera. It also suggests that there is an effect of altitude on the community structure and intergeneric relationships of soil AMF communities.

In addition to differences in distribution, AMF community diversity varies during different altitudes. Some studies have shown (Egan et al., 2017; Shi et al., 2019) that AMF community diversity increases and then decreases with increasing altitude, with different correlations at different altitude regions. Then, our results showed that AMF community diversity increases with altitude, unlike the results of other studies (Lugo et al., 2008; Guo et al., 2022). This may be due to the small study area, the low altitude and limited soil nutrients in this study. It has been shown that both increases in soil nutrients and decreases in anthropogenic disturbance positively affect AMF community diversity (Opik et al., 2008; Helgason and Fitter, 2009). In this study, soil nutrients increased significantly with altitude and were less disturbed by anthropogenic disturbances, which together with a variety of environmental factors influenced the AMF community, resulting in significant differences in community diversity during different altitudes. Therefore, the mechanisms by which different altitudes and vegetation in Helan Mountain affect the AMF community need to be further explored in future studies.

### 4.2. Influencing factors of soil arbuscular mycorrhizal fungi community structure

The soil physico-chemical properties in Helan mountain have an important influence on AMF community diversity, in agreement with the findings of many studies (Ma et al., 2021; Maitra et al., 2021). Besides, pH, WC, AP, OM, and AK were the main influencing factors to affect the diversity of the AMF community. Furthermore, pH and AP were highly significantly and negatively correlated with community diversity. It is possible that pH can affect not only the formation and development of AMF spores (Zhang et al., 2022a), but also indirectly by affecting plant community composition and plant uptake and utilization of soil nutrients (Xu et al., 2017). The effect of AP on AMF community diversity is consistent with the findings of Ji and Bever (2016) and Ceulemans et al. (2019), possibly because elevated AP levels inhibit AMF spore germination and mycelial growth and cause plants to reduce root symbiosis with AMF and take up P directly from the soil (Johnson et al., 2015; Shi et al., 2021). In addition, WC, OM, and AK were highly significantly and positively correlated with community diversity, most likely because that the increased WC not only promotes the reproductive development of AMF (Zhao et al., 2017), but also changes the selection and colonization of AMF by host plants, which has a significant effect on AMF community structure and diversity (Symanczik et al., 2015). OM could improve AMF reproduction to some extent (Zhao et al., 2022), and AK can promote AMF infestation of plants and affect AMF communities. Soil N was a minor factor affecting the AMF community, unlike the results of some other study (Ji and Bever, 2016), probably because the western slope of the Helan Mountain is gently sloping, making the variation of soil N between different altitudes insignificant.

The soil characteristics has an important influence on the AMF community, and the analysis of Heatmap (Figure 7) indicated that soil physicochemical properties had different effects on different genera of AMF. *Glomus*, as the dominant genus, was most affected by soil physicochemical properties and most genera were affected by soil factors, in agreement with some studies (Xu et al., 2017; Kim et al., 2022), explaining to some extent the differences in AMF communities between different altitudes. It further demonstrates that soil factors have an important influence on AMF community structure and diversity.

In addition, it has been shown that host plants are one of the main factors affecting AMF communities in montane ecosystems (Martinez-Garcia et al., 2015), which may be related to the fact that different vegetation has different nutrient requirements (Ezeokoli et al., 2020), and that plant species diversity has been found to be positively correlated with fungal community diversity in some studies (Johnson et al., 2003). In the middle and low-altitude regions (GR-CO), AMF communities differed less structurally and more in diversity, with higher plant diversity in UL (*Ulmus glaucescens* Forests) and SH (Shrub) plants and lower plant diversity in GR (Grass; Yang et al., 2022), the same variation as in AMF communities diversity, indicating the influence of plant diversity on AMF community diversity. The relative abundance of *Claroideoglomus* was higher in the mid and high altitude regions (PF-SU) than in the low-altitude regions, and *Claroideoglomus* was found to promote nutrient uptake by plants (Jamiolkowska et al., 2020), which may allow rising soil nutrients in the mid and high altitude regions to promote symbiosis between plants and *Claroideoglomus*, allowing plants to take up more nutrients and increase their biomass. It has been found that under more severe climatic factors at high-altitude, plants will select more for symbiosis with AMFs that can enhance plant cold resistance (Bauer et al., 2017; Dong et al., 2021). This may account for the marked differences in AMF community structure in the high-altitude region (PI, SU) compared to the low-altitude region. In terms of community genus level composition, the relative abundance of *unclassified_p_Glomeromycota*, *unclassified_o_Glomerales* and *unclassified_c_Glomeromycetes* was significantly higher in the high-altitude region and may be key genera for improving plant cold resistance. However, further studies are needed to determine the specific species and functions. In addition, there were also significant differences between the AMF community structures of PI and SU, and the community diversity of SU was much higher than that of PI, probably due to the significant differences in vegetation types between SU and PI, with SU having significantly higher plant diversity and soil nutrients than PI, and the fact that *Picea crassifolia* in PI was mainly symbiotic with ectomycorrhizal fungi and the AMF community was not a major fungal phylum (Wang and Han, 2022). It further demonstrates the influence of vegetation type and plant diversity on AMF communities.

In future research, the interrelationship between soil AMF communities and plant community characteristics and soil nutrients at different altitudes on the western slope of Helan Mountain can be investigated to further demonstrate the ameliorating effect of soil AMF communities on plants and soil, and to provide a theoretical basis for the protection of Helan Mountain ecology, vegetation restoration and soil management.

## 5. Conclusion

In the present study, soil AMF on the western slope of Helan Mountain belonged to one phylum, four class, six orders, 13 families, 18 genera and 135 species, with the dominant genus being *Glomus*. Soil AMF community structure and diversity differed significantly between different altitudes, with AMF community diversity increasing with increasing altitude. The physico-chemical properties of the soil and vegetation types have significant effects on the AMF community structure and diversity in soil, with soil pH, WC, AP, OM and AK being the main factors affecting the AMF community.

## Data availability statement

The original contributions presented in the study are included in the article/supplementary material, further inquiries can be directed to the corresponding author.

## Author contributions

PY: conceptualization, data curation, methodology, software, and writing-original draft preparation. HH: data curation, methodology, software, and writing-original draft preparation. YL: investigation. HZ: investigation and experimental help. JL: investigation. LS: investigation. QX: investigation. YL: investigation. JL: investigation. XN: investigation, supervision, funding acquisition, manuscript revising, and editing. All authors contributed to the article and approved the submitted version.

## Funding

This work was financially supported by the Key Project of Research and Development of Ningxia, China (Nos. 2020BFG03006 and 2021BEG02005), Project of Natural Science Foundation of Ningxia, China (No. 2020AAC03107), and Central Government Guides Local Science and Technology Development Project (No. 2022FRD05001).

## Conflict of interest

The authors declare that the research was conducted in the absence of any commercial or financial relationships that could be construed as a potential conflict of interest.

## Publisher’s note

All claims expressed in this article are solely those of the authors and do not necessarily represent those of their affiliated organizations, or those of the publisher, the editors and the reviewers. Any product that may be evaluated in this article, or claim that may be made by its manufacturer, is not guaranteed or endorsed by the publisher.

## Footnotes

^1^ http://drive5.com/uparse/
